# Climate hazards in Latin American cities: Understanding the role of the social and built environments and barriers to adaptation action

**DOI:** 10.1016/j.crm.2024.100625

**Published:** 2024

**Authors:** Anne Dorothée Slovic, Katherine Indvik, Lucas Soriano Martins, Josiah L. Kephart, Sandra Swanson, D. Alex Quistberg, Mika Moran, Maryia Bakhtsiyarava, Carol Zavaleta-Cortijo, Nelson Gouveia, Ana V. Diez Roux

**Affiliations:** a School of Public Health, University of São Paulo, São Paulo, Brazil; b Dornsife School of Public Health, Drexel University, Philadelphia, PA, United States; c Department of Preventive Medicine, University of Sao Paulo Medical School, São Paulo, Brazil; d Monash Sustainable Development Institute, Monash University, Melbourne, Australia; e School of Public Health, University of Haifa, Haifa, Israel; f Institute of Transportation Studies, University of California - Berkeley, CA, United States; g Unidad de ciudadanía intercultural y salud indígena, Facultad de Salud Pública y Administración, Universidad Peruana Cayetano Heredia, Lima, Peru

**Keywords:** Climate change, Climate hazards, Risk perception, Latin America, Urban, Adaptation

## Abstract

Climate hazards threaten the health and wellbeing of people living in urban areas. This study characterized reported climate hazards, adaptation action, and barriers to adaptation in 124 Latin American cities, and associations of climate hazards with urban social and built environment characteristics. We examined cities that responded to a global environmental disclosure system and that were included in the Urban Health in Latin America (SALURBAL) Project database.

The cities studied reported a median of three climate hazards. The most reported hazards were storms (61%) water scarcity (57%) extreme temperature (52%) and wildfires (51%). Thirty-eight percent of cities reported four or more distinct types of hazards. City size, density, GDP, and greenness were related to hazard reports, and although most cities reported taking actions to reduce vulnerability to climate change, 23% reported no actions at all. The most frequently reported actions were hazard mapping and modeling (47%) and increasing vegetation or greenspace coverage (45%). Other actions, such as air quality initiatives and urban planning, were much less common (8% and 3%, respectively). In terms of challenges in adapting to climate change, 35% of cities reported no challenges. The most frequently reported challenges were urban environment and development (43%) and living conditions (35%). Access to data, migration, public health, and safety/security were rarely reported as challenges. Our results suggest that climate hazards are recognized, but that adaptation responses are limited and that many important challenges to response action are not fully recognized.

This study contributes to understanding of local priorities, ongoing actions, and required support for urban climate vulnerability assessment and adaptation responses. Findings suggest the need for future research documenting local perceptions of climate hazards and comparison with documented climate hazards.

## 1 Introduction

Climate change is driving an increased frequency of extreme weather events, and long-term shifts in temperature and precipitation patterns. These changes have direct and indirect impacts on human health and the environment and generate enormous financial costs related to disaster response and recovery (Drakes et al., 2021). Extreme temperatures have been associated with over 5 % of all deaths in cities across Latin America (Kephart et al., 2022). Projected increases in the frequency and intensity of extreme temperatures and weather events will create water and food insecurity, threaten energy production, and drive greater transmission of vector-borne disease across the region (Hagen et al., 2022).

The severity of climate change impacts at the individual and population level is determined by hazard frequency and intensity, together with population exposure and vulnerability. Vulnerability to climate change is determined in part by social conditions and urban characteristics, which can mitigate or intensify health impacts (Birkmann et al., 2022). Across Latin America, fast-paced urbanization and population aging within the context of extreme social and economic inequality exacerbate vulnerability to climate-sensitive hazards and limit adaptive capacity, particularly in coastal areas (Nagy et al., 2019).

Responses to climate change have been developed at the national level throughout the Latin American region, yet existing national, regional, and global commitments to mitigation and adaptation action are recognized as insufficient (IPCC, 2018, Parry et al., 2019). Within this context, sub-national action plays an increasingly critical role in effectively responding to both current and longer-term change. Local-level, context-specific actions can be more direct and flexible, and cities and local governments are increasingly leveraging the power of cross-sectoral networks to implement local-level interventions, seek funding, and in turn drive more ambitious commitments at the national and global levels (Sharifi, 2021, Solecki et al., 2018).

Public support is key to achieving required behavioral change and ensuring the effectiveness and sustainability of climate action, as well as for driving policy change. Across Latin America, most residents view climate change as a major threat, although this perception varies by country (Watts et al., 2015). Adaptive behavior has been positively associated with risk perception (van Valkengoed and Steg, 2019), suggesting that increasing people’s and groups’ understanding of climate hazards may increase motivation for and willingness to engage in adaptive behavior (Houser et al., 2022; Kunreuther and Slovic, 2021; Musacchio et al., 2021). Policymakers and local stakeholders do not always agree regarding the severity of climate risk, and studies on the factors determining risk perception and motivating adaptation action tend to focus on individual case studies (Dieckmann et al., 2021, Frank et al., 2011, Lawrence et al., 2014, Lemée et al., 2022).

Associations have also been documented between characteristics of the urban built and social environment and vulnerability to climate hazards. Densely built-up and populated city centers usually experience higher air temperatures than city peripheries because of the urban heat island effect, exacerbating exposure to heat among residents in these areas (Zhao et al., 2014). Heat exposure may also be amplified by individuals’ socioeconomic characteristics, and high-income inequality has been associated with greater excess mortality from extreme cold, especially among older adults. Segregation and poverty are associated with higher excess mortality due to cold (Bakhtsiyarava et al., 2023).

The results for heat-related mortality require further investigation. For example, low-income urban populations residing in areas with high density and limited greenspace in Thailand showed an increased likelihood of experiencing heat stress (Arifwidodo and Chandrasiri, 2020). In Brazil, income inequality and spatial segregation have been shown to exacerbate vulnerability, with lowest-income residents forced to settle in areas prone to frequent environmental hazards such as floods (Rasch, 2017). Socioeconomic inequality in Brazilian cities has been associated with increased climate risk and death tolls from extreme events (Travassos et al., 2021).

Urban practitioners’ and policymakers’ understanding of and response to climate hazards can have important effects on defining national and global climate action (Young et al. 2022). The ways governments communicate risks and levels of trust in public institutions trust have a direct impact on risk perception, changes in behavior, and climate hazards preparedness, which in turn can positively or negatively impact climate adaptation action plans (Lee et al., 2015; Smith&Mayor 2018; Wachinger et al. 2020). Work documenting risk perception and exploring the connections between the built and social environments and perceived risk and vulnerability to climate hazards remains limited (Azócar et al., 2021). The goal of this analysis was to 1) explore the prevalence of perceived climate hazards, adaptation actions, and barriers to adaptation action as reported by city officials, and 2) to examine the association between these perceived and projected climate hazards and characteristics of the urban built and social environment in Latin American cities. Understanding the types of hazards cities identify, what actions they are taking to respond or prepare, any barriers to this action, and how urban characteristics are related to risk perception can inform mitigation and adaptation strategies throughout Latin America and globally.

## 2 Material and methods

### 2.1 Data sources

Data were extracted from the CDP (formerly known as the Carbon Disclosure Project) 2019 City Questionnaire and the Urban Health in Latin America (or SALURBAL) Project. CDP is an international non-profit organization that coordinates a global environmental emissions disclosure system for subnational governments and private sector actors in over 80 countries. Operating under a theory of change that believes that “you can't manage what you don't measure,” CDP conducts annual surveys to track multiple domains including reported emissions, vulnerability, and adaptation and mitigation actions. Data are self-reported via an online portal, and responses for cities are not independently audited. According to our collaborators at CDP, questionnaires are often completed by sustainability or resilience officers within municipal government administrations, and CDP engagement officers in each region provide support to cities throughout the reporting process. Each year, CDP presents overall scoring for cities and companies to highlight progress and incentivize more ambitious and transparent climate action. In 2019, CDP received a record number of responses from cities across Latin America.

The SALURBAL Project has developed an unprecedented data resource on urban environments and health outcomes for all urban agglomerations of 100,000 residents or more across 11 countries in Latin America. The project compiles and harmonizes pre-existing data on a range of factors including demographic characteristics, mortality, health behaviors and risk factors, and social and built environment attributes from Latin American cities, countries, and regional institutions (Quistberg et al., 2019).

By selecting cities included in both datasets, we were able to explore associations between the hazards and actions reported to CDP and other urban characteristics included within the SALURBAL data resource.

### 2.2 CDP cities 2019 Questionnaire and outcome variables

We selected a subset of categorical questions from Section 2: *Climate hazards and vulnerability* and Section 3: *Adaptation actions* of the CDP Cities 2019 Questionnaire (See Table 1)*.* See https://www.cdp.net/ for the full text of included questions, response formats, and response options. This was the last questionnaire administered and processed prior to the onset of the COVID-19 pandemic, after which substantial changes took place regarding survey administration and the questionnaire itself. Additionally, many of the indicators available within the SALURBAL data resource are available for the years 2018–2019 (See Section 2.4.).

**Table 1:** Selected questions, variables, and response options from the CDP Cities 2019 Questionnaire. ^1^Response options were defined by CDP, with survey respondents invited to select all responses that apply to each question. The above response options represent categories created by the authors to simplify analysis. A given respondent may have selected responses that fall into one or more of these categories. For a list of all responses and their categorization, please refer to Appendix A.

**Question**	**Variable of interest**	**Response options^1^**
Section 2, Question 1 (2.1): Please list the **most significant climate hazards faced by your city** and indicate the probability and consequence of these hazards, as well as the expected future change in frequency and intensity.	Climate hazards	Biohazard Chemical change Extreme temperature Flood Landslide Storm Water scarcity Wildfire
3.0 Please describe the **main actions you are taking** to reduce the risk to, or vulnerability of, your city’s infrastructure, services, citizens, and businesses from climate change as identified in the Climate Hazards section.	Adaptation response actions	Climate risk planning and management Education, awareness, and engagement Flood management Hazard mapping and modeling Health-related actions Increasing vegetation/greenspace coverage Infrastructure improvement Air quality initiatives Monitoring Nature-based solutions Temperature management Urban planning Water management (No action)
2.2: Please identify the **factors that most greatly affect your city’s ability to adapt** to climate change and indicate how those factors either support or challenge this ability.	Challenges to adaptation action	Access to quality/relevant data Community engagement Financial/resource availability Migration Public health Safety and security Governance Living conditions Financial/ resources availability Education and services Urban environment and development

Climate hazards: We reviewed climate hazards reported by Latin American cities in response to survey Question 2.1 and categorized similar hazards into eight groups: biohazard, chemical change, extreme temperature, flood, landslide, storm, water scarcity, and wildfire. This categorization was informed by a review of primary global climate hazards (Watts et al., 2021) and consultation with CDP survey administrators.

Adaptation actions: Cities were asked to identify any actions aimed “to reduce the risk to, or vulnerability of” their “infrastructure, services, citizens and business from climate change.” We grouped the 41 individual actions reported by cities into 13 action categories, based on available climate adaptation literature and in consultation with CDP representatives.

Challenges to adaptation action: Finally, cities were asked to “identify the factors that most greatly affect your city’s ability to adapt to climate change and indicate how those factors either support or challenge this ability.” Our analysis focused on adaptation challenges, and we grouped the 25 types of challenges reported into 11 categories, again in consultation with CDP representatives.

### 2.3 Sample selection

In total, 304 cities from 15 countries in Latin America responded to the 2019 questionnaire. Of these, 184 cities from nine countries corresponded to urban areas included in the Urban Health in Latin America (SALURBAL) Project. SALURBAL defines cities as administrative units or clusters of administrative units (e.g., municipalities) that encompass the built-up area of urban agglomerations. The project also collects data for smaller administrative areas (e.g., *municipios, comunas, distritos*). (For additional details, please see: https://drexel-uhc.github.io/salurbal-city-selection-scrolly/) Most of the CDP survey respondents included in this analysis correspond to these smaller units and hence do not encompass the full urban agglomeration identified as a “city” in SALURBAL; for simplicity throughout this paper, we refer to each administrative unit reporting to CDP as a CDP city.

**Fig. 1:**
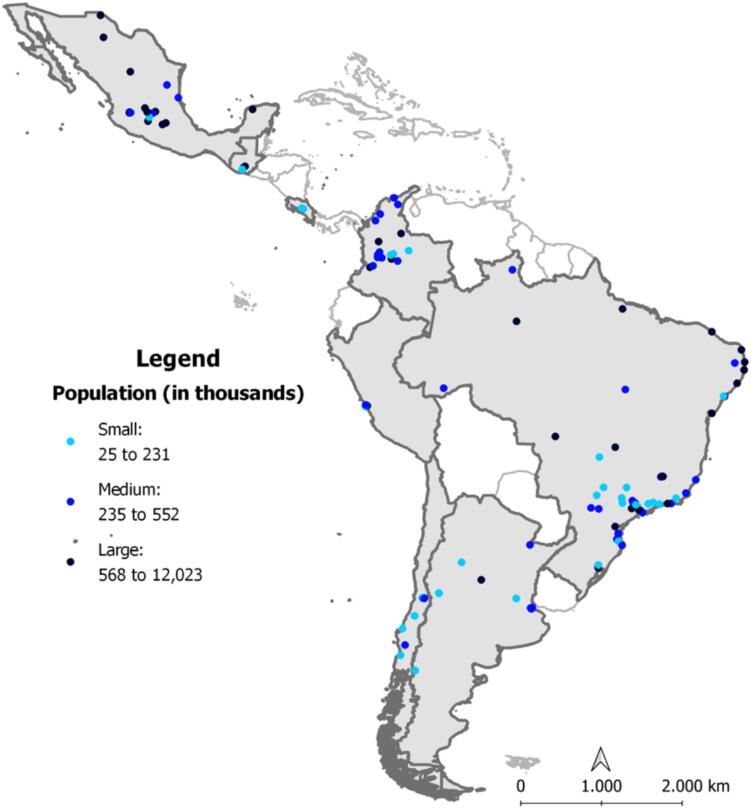
Cities responding to the CDP Climate Change 2019 Questionnaire and included in the SALURBAL data resource, by population tertile. Some CDP cities represent subsets of the larger urban agglomerations included in the SALURBAL Project.

We excluded 24 incomplete surveys, then reviewed the remaining 160 to establish matches to SALURBAL areas. Matching criteria included name, georeferenced location, land area (in square km), and population. For each city, we compared these values as reported to CDP with the values available in the SALURBAL data resource. A minimum agreement of 90 % in surface area and 95 % population was required for inclusion. Descriptions of administrative boundaries as reported to CDP were reviewed to confirm apparent matches and correct for survey user error (e.g., misplaced decimal points and other obvious typos). Our final selection included 124 CDP cities from eight countries in Latin America: Argentina: 11 cities, Brazil: 54, Chile: 10, Colombia: 17, Costa Rica: 3, Guatemala: 3, Mexico: 17, Peru: 12. Fig. 1 shows selected cities by population size. In those cases CDP where cities are only subsets of the larger urban agglomerations included in SALURBAL, data from the appropriate smaller area was used in analyses.

### 2.4 City-level characteristics

We selected a set of city characteristics from the SALURBAL project data resource for exploration as potential correlates of reported climate hazards. These variables were selected based on their documented connections to climate vulnerability, and on their availability (coverage) for our area of study (See Table 2).

**Table 2:** Selected physical and social environment characteristics of Latin American cities.

**Physical environment**	**Variable description**	**Interpretation**	**Source(s)**
Greenness	Zonal median of annual maximum Normalized Difference Vegetation Index (NDVI) in an area, water excluded from satellite data (describe from BEC data)	A higher value indicates higher level of greenness.	(Ettehadi Osgouei et al., 2019, NASA, xxxx)
Population density	Population per square kilometer of all the built-up area inside the geographic boundary calculated using population (see below) and total urban area of the city from Global Urban Footprint in 2012.	A higher value indicates a denser urban development pattern.	(Esch et al., 2018, Esch et al., 2017, Esch et al., 2011)
City fragmentation (i.e., patch density)	Number of urban patches (areas of contiguous built-up area) divided by total area (square kilometers) calculated using built-up areas identified from satellite imagery.	Higher patch density indicates greater fragmentation of urban expansion. Fragmentation is an indicator of urban sprawl.	(Irwin and Bockstael, 2007, McGarigal et al., 2012, Weisstein, 2012)
**Social environment**	**Variable description**	**Interpretation**	**Source**
Population (2016)	Total population within the geographic boundary as calculated from census or official government population projections.	A higher value indicates more people reside within the geographic unit.	Estadística y Censos (INDEC)Brazil: Ministério da Saúde website (https://datasus.saude.gov.br/populacao-residente) Chile: Instituto Nacional de Estadistica (INE) website (https://www.ine.cl/estadisticas/sociales/demografia-y-vitales/proyecciones-de-poblacion)Colombia: Departamento Administrativo Nacional de Estadísticas (DANE) website (https://www.dane.gov.co/index.php/estadisticas-por-tema/demografia-y-poblacion/proyecciones-de-poblacion)Costa Rica: Instituto Nacional de Estadística y Censos (INEC) Guatemala: Instituto Nacional de Estadística (INE)Mexico: Instituto Nacional de Estadística, Geografía e Informática (INEGI) and the Consejo Nacional de Población (CONAPO)Peru: Instituto Nacional de Estadística e Informática (INEI)
Population educational attainment score	Educational achievement measured as the sum of z-scores of 1) percent of the population aged 25 or above who completed high school or more and 2) percent of population aged 25 or above who completed university level or reported in the most recent census of the country.	Higher score signifies greater educational achievement in the population. For details, see Ortigoza, et al., 2019.	(Ortigoza, et al., 2021)
Per-capita GDP	Annual subnational GDP per capita for each SALURBAL city, most often for departments. (Note: Only national level is available for Costa Rica.)	Higher values indicate greater purchasing power parity, represented in constant 2011 international USD.	(Kummu et al., 2018)

### 2.5 Data analysis

We determined the frequency of climate hazards, actions, and challenges reported by each CDP city, as well as hazard distribution by country. We then ran chi-square tests to examine potential associations between the types of climate hazards reported and selected city characteristics. Given our sample size and the nature of the indicators included (continuous, heavily skewed and/or with irregular distribution), each built or social environment characteristic was divided into tertiles (low, medium, high). All analyses were conducted in SAS/STAT® v9.4 (SAS Institute Inc., Cary, NC, USA), using a cut-off of p < 0.05 for statistical significance.

## 3 Results

The following sections describe our findings regarding the prevalence of climate hazards reported for Latin American cities, associations between these hazards and urban built and social environment characteristics, adaptation actions, and challenges to action.

### 3.1 Climate hazards

In total, 124 Latin American cities reported 32 climate hazards. The seven most reported hazards by CDP cities in Latin America were (Table 3): storm (61 % of all cities reporting), water scarcity (57 %) extreme temperature (52 %) and wildfires (51 %) followed by landslides (44 %), floods (30 %), and biohazards (27 %). Cities reported between one and seven distinct hazards, with an average of 3.1 hazards reported per city (median of 3). Twenty-three cities (18 %) reported only one hazard, 31 (25 %) reported two hazards, 24 (19 %) reported three hazards, and 46 (37 %) reported four or more distinct climate hazards. Table 3 presents the number of hazards and types of hazards reported by cities in each country.

**Table 3:** Percent of CDP cities by reporting significant climate hazards faced as of 2019. AR-Argentina; BR-Brazil, CL- Chile; CO-Colombia; CR- Costa Rica; GT-Guatemala; MX- Mexico; PE-Peru.

**Hazard**	**All cities**	**AR**	**BR**	**CL**	**CO**	**CR**	**GT**	**MX**	**PE**
	(N = 124)	(N = 11)	(N = 54)	(N = 10)	(N = 17)	(N = 3)	(N = 3)	(N = 17)	(N = 9)
**Average number of hazards reported**	3.1	3.2	3.4	3.4	3.6	2.3	3.7	2.3	2.2
**More than 3 hazard groups reported (%)**	38 %	36 %	44 %	40 %	47 %	33 %	67 %	18 %	11 %
**Storm (%)**	61 %	91 %	61 %	50 %	47 %	100 %	100 %	71 %	22 %
**Water scarcity (%)**	57 %	55 %	43 %	60 %	41 %	33 %	33 %	24 %	78 %
**Extreme Temperature (%)**	52 %	73 %	50 %	80 %	41 %	33 %	67 %	59 %	89 %
**Landslide (%)**	44 %	0 %	39 %	20 %	59 %	0 %	67 %	12 %	0 %
**Wildfire (%)**	51 %	0 %	33 %	30 %	59 %	0 %	33 %	12 %	0 %
**Flood (%)**	30 %	55 %	61 %	50 %	71 %	33 %	33 %	29 %	22 %
**Biohazards (%)**	27 %	45 %	48 %	50 %	47 %	33 %	33 %	24 %	11 %

**Fig. 2:**
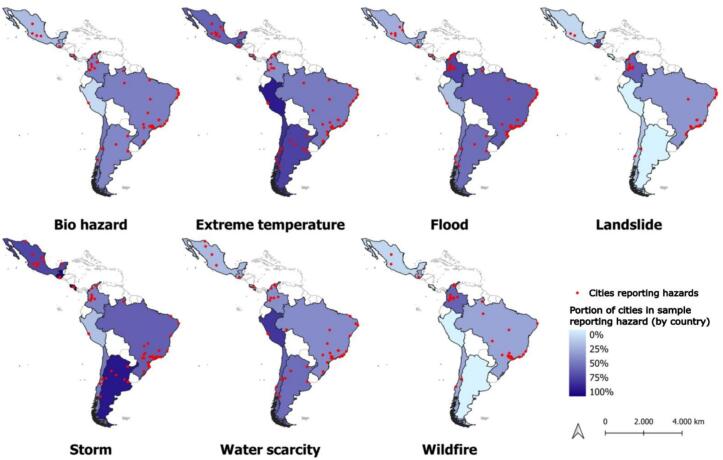
**Cities responding to the CDP questionnaire and included in the SALURBAL Project database.** Red dots indicate reports of specific hazards, and shading indicates the portion of cities in each country that reported a given hazard. Note overlapping red dots represent responses received from multiple municipalities within major metropolitan areas, including Mexico City, Bogotá, Lima, Santiago, and São Paulo. (For interpretation of the references to colour in this figure legend, the reader is referred to the web version of this article.)

Most CDP cities (61 %) reported storms, and storms were reported by the majority of cities (>50 %) in Argentina, Brazil, Costa Rica, Guatemala, and Mexico, though storms were notably absent in reports from Peruvian cities (Fig. 2). Water scarcity was most frequently reported by cities in Argentina, Chile, and Peru; these countries also had the largest portion of cities reporting extreme temperatures as hazards, and the smallest portion of cities reporting landslide hazards. Most Colombian cities reported landslides, wildfires, and floods. Cities reporting floods tended to be concentrated along the coast, except for cities in Colombia where a higher portion of inland cities reported flood hazards. Biohazards were reported by cities in Argentina, Brazil, Chile, and Colombia, but less frequently by cities in Peru and Mexico.

### 3.2 Reported hazards in relation to city built and social environment characteristics

Table 4 shows associations of reported hazards with city characteristics. Small cities (231,000 residents or fewer) were less likely than medium or large cities to report landslides, floods, and biohazards. They were also less likely to report more than three hazards (29 % vs. 43 % vs. 51 % for small, medium, and large cities, respectively). Less dense cities were less likely than denser cities to report extreme temperatures, landslides and floods, and were also less likely to report more than three hazards than higher density cities. CDP cities with higher GDP tended to report fewer landslides (p = 0.06). CDP cities with higher education levels tended to report more extreme temperatures. Greener cities reported less water scarcity and extreme temperatures, but more landslides. More fragmented cities reported more storms and landslides but less water scarcity.

**Table 4:** Percent of cities reporting climate hazards by city characteristic. *Minimum and maximum values are listed for each tertile. **Higher educational achievement score values signify better educational achievement in the population.

**Measure**	Group/tertile*	**Storm**	**Water scarcity**	**Extreme Temperature**	**Landslide**	**Wildfire**	**Flood**	**Biohazard**	**More than 3 (median)**
**Population (in thousands)**	**Small** **(25, 231)**	54 %	49 %	49 %	12 %	24 %	32 %	20 %	29 %
	**Medium** **(235, 552)**	71 %	48 %	52 %	40 %	31 %	69 %	50 %	43 %
	**Large** **(568, 12,023)**	59 %	37 %	71 %	37 %	27 %	56 %	54 %	51 %
	**P-value**	0.228	0.4701	0.0976	**0.0098**	0.7947	**0.0026**	**0.0026**	**0.009**
**Population density (thousands per square km)**	**Small** **(2.43, 5.56)**	68 %	44 %	44 %	17 %	34 %	34 %	29 %	24 %
	**Medium** **(5.59, 9.560)**	64 %	43 %	57 %	29 %	21 %	60 %	43 %	38 %
	**High** **(9.565, 28.4)**	51 %	46 %	71 %	44 %	27 %	63 %	51 %	51 %
	**P-value**	0.2517	0.9478	**0.049**	**0.0288**	0.4281	**0.0156**	0.1251	**0.0435**
**GDP per capita in 2010 USD thousands**	**Low** **0.9, 13.1**	51 %	37 %	63 %	34 %	27 %	56 %	37 %	42 %
	**Medium** **13.3, 22.0**	73 %	46 %	46 %	39 %	29 %	51 %	46 %	39 %
	**High** **22.2, 36.2**	60 %	50 %	62 %	17 %	26 %	50 %	40 %	33 %
	**P-value**	0.1196	0.4469	0.2229	0.064	0.9467	0.8417	0.6646	0.7352
Educational achievement score**	**Low** **−2.50, −0.21**	63 %	50 %	45 %	35 %	28 %	55 %	30 %	33 %
	**Medium** **−0.197, 0.918**	51 %	32 %	54 %	29 %	29 %	46 %	49 %	49 %
	**High** **0.919, 7.97**	68 %	53 %	75 %	28 %	28 %	58 %	45 %	43 %
	**P-value**	0.3073	0.12	**0.0199**	0.7482	0.9792	0.5717	0.1941	0.6455
**Greenness (NDVI index)**	**Low** **0.15, 0.65**	63 %	61 %	71 %	15 %	15 %	49 %	44 %	39 %
	**Medium** **0.66, 0.818**	67 %	43 %	60 %	33 %	38 %	55 %	43 %	45 %
	**High** **0.822, 0.92**	54 %	29 %	41 %	41 %	29 %	54 %	37 %	29 %
	**P-value**	0.4502	**0.0149**	**0.0259**	**0.0245**	0.0538	0.8456	0.7666	0.1786
**Fragmentation**	**Low** **0.019, 0.308**	46 %	59 %	54 %	15 %	32 %	39 %	27 %	29 %
	**Medium** **0.316, 0.648**	64 %	31 %	55 %	38 %	26 %	55 %	50 %	36 %
	**High** **0.674, 1.21**	73 %	44 %	63 %	37 %	24 %	63 %	46 %	49 %
	**P-value**	**0.0396**	**0.0407**	0.6191	**0.0336**	0.7409	0.0809	0.1251	0.1786

### 3.3 Adaptation actions to reduce vulnerability

Overall, CDP cities reported an average of 1.9 actions to reduce risk or vulnerability to climate change o (Table 5, median: 1 action per city). The most frequently reported actions were hazard mapping and modeling (47 %); increasing vegetation or greenspace coverage (45 %); education, awareness, and engagement activities (35 %); climate and risk management (32 %); and water management (30 %). The least frequently reported were air quality initiatives (8 %) temperature management (4 %), urban planning (3 %), and nature-based solutions (2 %). Twenty-eight (23 %) cities reported no actions, while nineteen (15 %) cities reported more than three climate adaptation actions, with a maximum of twelve actions reported by a single city.

**Table 5:** Percent of cities reporting climate adaptation actions, overall and by country. AR-Argentina; BR-Brazil, CL- Chile; CO-Colombia; CR- Costa Rica; GT-Guatemala; MX- Mexico; PE-Peru.

**Action group**	**Total** **(N = 124)**	**AR** **(N = 11)**	**BR** **(N = 54)**	**CL** **(N = 10)**	**CO** **(N = 17)**	**CR** **(N = 3)**	**GT** **(N = 3)**	**MX** **(N = 17)**	**PE** **(N = 9)**
**Climate risk planning and management**	32 %	45 %	28 %	30 %	71 %	0 %	67 %	18 %	0 %
**Education, awareness, and engagement**	35 %	27 %	17 %	70 %	76 %	33 %	0	29 %	67 %
**Flood management**	10 %	9 %	13 %	30 %	0	0	0	6 %	0
**Hazard mapping and modeling**	47 %	18 %	54 %	50 %	59 %	33 %	67 %	53 %	0
**Health-related actions**	17 %	0	26 %	20 %	12 %	33 %	0	12 %	0
**Increasing vegetation/greenspace coverage**	45 %	64 %	33 %	70 %	29 %	0 %	67 %	47 %	100 %
**Infrastructure improvement**	16 %	36 %	13 %	20 %	18 %	0 %	33 %	12 %	11 %
**Air quality initiatives**	8 %	0	7 %	10 %	12 %	0	0	18 %	0
**Monitoring**	23 %	18 %	26 %	40 %	12 %	0	0	29 %	11 %
**Nature-based solutions**	2 %	0	4 %	0	0	0	0	0	0
**Temperature management**	4 %	0	0 %	10 %	6 %	0	0	6 %	22 %
**Urban planning**	3 %	0	4 %	0	6 %	33 %	0	0	0
**Water management**	30 %	27 %	19 %	60 %	6 %	0	33 %	53 %	78 %
**No action**	23 %	0	11 %	10 %	94 %	0	0	24 %	11 %

### 3.4 Challenges to climate adaptation action

Table 6 shows challenges reported by CDP cities in their ability to adapt to climate change. Overall, cities reported an average of 1.1 challenges (median 1 challenge). The most frequently reported challenges were urban environment and development (43 %), living conditions (35 %), education and services (26 %), and financial and resources (25 %). The least frequently reported challenges were community engagement (8 %), access to quality/relevant data (4 %), migration (4 %), public health (2 %) and safety and security (2 %). Forty-five (36 %) cities reported no challenges while 46 (37 %) reported more than three challenges.

**Table 6:** Percent of CDP cities reporting challenges to climate change adaptation.

	**All cities**	**AR**	**BR**	**CL**	**CO**	**CR**	**GT**	**MX**	**PE**
**Challenge groups**	**(N = 124)**	**(N = 11)**	**(N = 54)**	**(N = 10)**	**(N = 17)**	**(N = 3)**	**(N = 3)**	**(N = 17)**	**(N = 9)**
**Access to quality/relevant data**	4 %	0	2 %	20 %	6 %	0	0	6 %	0
**Community engagement**	8 %	9 %	4 %	0	6	0	0	24 %	22 %
**Financial/resource availability**	25 %	0	19 %	50 %	29 %	33 %	0	41 %	33 %
**Migration**	4 %	0	2 %	0	18	0	0	6 %	0
**Public health**	2 %	0	2 %	0	6	0	0	0	0
**Safety and security**	2 %	0	4 %	0	0	0	0	6	0
**Governance**	13 %	0	9 %	0	35 %	0	3 %3	12 %	22 %
**Living conditions**	35 %	27 %	39 %	30 %	24 %	100 %	0	41 %	22 %
**Economic**	17	9 %	20 %	0	35 %	0	33 %	12 %	0
**Education and services**	26 %	36 %	22 %	0	29 %	0	0	59 %	11 %
**Urban environment and development**	43 %	27 %	41 %	90 %	24 %	67 %	0	59 %	33 %

## 4 Discussion

This study examined the climate hazards, adaptation action, and barriers to adaptation reported by 124 Latin American cities. Overall, cities identified a median of 3 climate hazards. The most reported hazards were storms (61 %) water scarcity (57 %) extreme temperature (52 %) and wildfires (51 %). Thirty-eight percent of cities reported four more distinct types of hazards. CDP cities also reported taking actions to reduce vulnerability to climate change, but 23 % reported no actions at all. The most frequently reported actions were hazard mapping and modeling (47 %) and increasing vegetation or greenspace coverage (45 %). Other actions, such as air quality initiatives and urban planning, were much less common (8 % and 3 %, respectively). In terms of challenges in adapting to climate change, 35 % of cities reported no challenges at all. The most frequently reported challenges were urban environment and development (43 %) and living conditions (35 %). Surprisingly, access to data, migration, public health, and safety/security were rarely reported as challenges (all reported by fewer than 4 % of cities). Overall, our results suggest that climate hazards are recognized, but that adaptation responses are limited and that many important challenges to response action are not fully recognized.

It is not surprising that storms were the most prevalent hazard reported given their pervasiveness, impacts, and connections to climate change. Extreme temperature and wildfires have been found to exacerbate environmental degradation such as water shortage and drought, loss of habitat and biodiversity and air pollution (Birnbaum et al., 2022, Braun et al., 2021, Desbureaux and Rodella, 2019). Prior research suggests that within the Latin American region these hazards have impacted health negatively, increasing the spread of infectious diseases, affecting birth weight, and increasing mortality risks due to extreme temperatures (Bakhtsiyarava et al., 2023, Kephart et al., 2022). Other negative impacts associated with these reported hazards include forced food security, migrations, conflicts and violence, and economic and political instability (Franco et al., 2022, Grasa, 2020). There was some geographic patterning of the hazards reported. For example, mountainous cities frequently reported landslide hazards. This may reflect both geographic characteristics (e.g., steep slope and soil type) and the prevalence of informal settlements in these areas (Puente-Sotomayor et al., 2021). The lack of storms reported by Peruvian cities is likely explained by the concentration of CDP cities within the Lima metropolitan area located in a subtropical desert climate. The high proportion of coastal cities reporting flood hazards is reasonable given their geography.

We also sought to better understand associations between climate hazards and urban social and built environment characteristics and found that some city characteristics were related to the hazards reported. For example, greener cities reported less water scarcity and extreme temperatures but more landslides. This could be due, in part, to the potential benefits associated with the presence of urban greenspaces in reducing temperature by mitigating the heat island effect and reducing water scarcity by managing water runoff in cities (Rahman et al., 2020, Reis and Lopes, 2019, Wang et al., 2021). Floods and landslides were more frequently reported by medium and large cities. These findings may resonate with documented flooding hazards in large coastal cities across the region, where 20 megacities are at high risk of sea-level rise (Griggs and Reguero, 2021). In addition, large cities were more likely than smaller cities to report multiple hazards. This could be linked to their geographic location or to greater awareness of leaders of these cities of environmental hazards linked to climate change.

The most frequently reported hazards (storms and water scarcity) can result in both immediate threats to (e.g., housing and sanitation challenges) and longer-term impacts on human health (e.g., disruptions to agricultural production and food security; increased range, and transmission of infectious diseases), and can drive additional, less direct health conditions (e.g., post-traumatic stress disorder and depression). CDP cities often reported multiple, overlapping hazards, which will continue to create challenges for both emergency response and permanent critical infrastructure and will require complex adaptive responses.

Our review of reported adaptation actions revealed substantial variation across included cities. Notably, 23 % of CDP cities reported no adaptation actions at all. Efforts to identify, categorize, and assess the efficacy of adaptation actions are increasing across research and development sectors, though critical evidence gaps remain. That the most frequently reported actions related to hazard mapping and modeling may be explained in part by efforts over recent decades to build technical capacity within the region, where adaptation actions related to capacity building, planning and management, and behavioral change have been increasingly common (Biagini et al., 2014). Nevertheless, actions related to education, awareness and engagement were relatively limited in our sample (only 35 % of cities).

Very few CDP cities reported adaptation actions related to urban planning, despite the well-established role of urban planning tools in mainstreaming climate adaptation, particularly in low-income areas (Núñez Collado and Wang, 2020, Runhaar et al., 2018). At the same time, cities frequently reported living conditions and development patterns as barriers to adaptation. Air quality initiatives were very infrequently reported despite well documented air quality challenges in the region (Gouveia et al., 2019). These results suggest opportunities for improving adaptation actions and supporting cities in planning and implementing them (at least as reported). This is consistent with Ryan and Bustos (2019) who highlight knowledge deficits and lack of long-term vision within the region related to adaptation initiatives.

Our sample of CDP cities most frequently cited urban environment and development factors, socioeconomic factors related to living conditions and urban services, and resource availability as key challenges to adaptation action. This categorization aligns to some extent with the most prevalent challenges to adaptation observed across the global south, which include behavioral barriers and financial and human resource limitations (Piggott-McKellar et al., 2019). Numerous studies have documented the role of available resources in determining the types and ambition of adaptation action that are undertaken and prioritized by local governments (Kim and Grafakos, 2019, Leal Filho et al., 2019, Lesnikowski et al., 2013). In our sample, larger cities were more likely to report more climate hazards. Globally, the most documented barriers to national adaptation policy relate to financial resources, institutional fragmentation, and information dissemination (Lee et al., 2022).

Robust institutions and effective governance are key to identifying, prioritizing, and responding to climate hazards, and barriers related to institutional capacity, extreme inequality, corruption, and mistrust in government are common across Latin America. Despite this context, only 12 % of CDP cities reported governance as a barrier to adaptation; this may reflect the limitations of self-reporting. It might also reflect challenges faced by smaller local authorities when proposing climate action within the context of larger urban administrations. Notably, most of the cities did not cite issues with access to quality and relevant data as a challenge to adaptation, despite well-documented data gaps in the region (related to local-level change and impacts/outcomes). Cities may be less likely to prioritize efforts to improve data collection and monitoring systems if these limitations are not explicitly identified. Public health was also infrequently identified as a challenge to adaptation. Future work might explore post-pandemic public health risks and possible barriers to adaptation, and the ways pandemic responses to public health questions were reported to CDP. Notably, 36 % of cities reported no challenges to adaptation at all.

This study relies on self-assessments of climate risk and impacts, which implies both strengths and limitations. The CDP team provides technical support to public officials completing the annual survey in order to improve standardization of the criteria applied by survey respondents. Perception is nevertheless subjective, and the characteristics of individual survey respondents (e.g., time in office, professional background, and personal experience and vulnerability) undoubtedly create challenges for interpreting these results. It is possible that individual respondents’ level of knowledge of these issues in part determines the number and nature of the hazards, actions, and challenges they identify, which may not reflect city leadership at large.

CDP surveys are extensive and contain sections on topics ranging from air pollution to food systems to transport. Depending on their familiarity with the topic, city officials may or may not associate climate change and related hazards to specific management challenges and may or may not draw connections to safety and security. Disclosure relies on self-reporting, and responses are scored by CDP and reported back with feedback and recommendation to each city. While scores can be kept private upon a city’s request, most disclosure to CDP is public, and anyone can access the open-source Open Data Platform.

Important challenges also arise when making cross country comparisons of secondary data. As documented by Quistberg et al. (2019), the SALURBAL team has developed a rigorous protocol for compiling and harmonizing data on social, economic, environmental, and health outcomes for nearly 400 cities and 1436 sub-geographies across Latin America to support meaningful region-wide assessments.

Additional limitations to be considered include the outdatedness of 2019 survey data, in part due to pandemic delays and subsequent changes to survey administration and questionnaire content. We hope that by providing a snapshot of cities’ reports, this work will support future assessments of the ways these perceptions are evolving over time.

Finally, the selection of cities necessarily reflects a “self-selection” of cities who engaged with the CDP initiative and is further limited to the cities included within the SALURBAL data resource. For this reason, the number of cities was examined as a portion of those reporting overall within each country, and we do not draw any conclusions regarding the portion of cities reporting overall.

Despite these limitations, included responses represent a large, diverse set of city governments and provide insight regarding the existing knowledge and priorities of local authorities across multiple countries in the region.

## 5 Conclusions

Latin American cities in our sample reported multiple climate related hazards, but reports of adaptation actions were generally less frequent. In 2019, CDP cities reported an average of three hazards each, with most cities reporting at least two climate hazards. Several built and social environment factors may play a role in predicting cities’ identification of climate hazards. Specifically, city greenness, population density, population size, and city fragmentation emerged as predictors of hazard reporting and may inform the types of response actions developed by cities, though more research on these connections is needed. About a third of CDP cities reported no adaptation actions, and urban planning and air quality initiatives were infrequently identified. In the years before the COVID-19 pandemic, data availability and public health challenges were rarely cited as barriers to adaptation action for cities in Latin America.

This study contributes to our understanding of the way city officials across Latin America perceive and respond to climate risk and supports the need for further research exploring the role of city characteristics in predicting climate risk perception and responses. Comparing perceived risk to observed hazards and impacts may shed light on gaps in local officials’ understanding of climate risk, and support knowledge to policy translation for improved urban planning. Taking stock of the types of adaptation actions that are or are not prioritized, as well as barriers to this action, may support the direction of available resources and capacity building efforts to ensure that climate action effectively overcomes these barriers. As cities and local governments increasingly assume a leading role in climate action, improved understanding of risk perception, adaptation priorities, and challenges faced will inform efforts to advance this critical work. By drawing attention to the extensive co-benefits of bold climate action, subnational leaders might capitalize on the potential to secure increased political, public, and financial support to deliver and accelerate their climate action plans.

## CRediT authorship contribution statement

**Anne Dorothée Slovic:** Writing – review & editing, Writing – original draft, Project administration, Formal analysis, Data curation, Conceptualization. **Katherine Indvik:** Writing – review & editing, Writing – original draft, Methodology, Formal analysis, Conceptualization. **Lucas Soriano Martins:** Methodology, Formal analysis, Data curation. **Josiah L. Kephart:** Writing – review & editing, Writing – original draft, Visualization, Validation. **Sandra Swanson:** Visualization, Validation, Conceptualization. **D. Alex Quistberg:** Writing – review & editing, Writing – original draft, Visualization, Data curation. **Mika Moran:** Visualization, Conceptualization. **Maryia Bakhtsiyarava:** Visualization, Methodology. **Carol Zavaleta-Cortijo:** Visualization, Conceptualization. **Nelson Gouveia:** Writing – review & editing, Writing – original draft, Visualization, Validation, Supervision, Conceptualization. **Ana V. Diez Roux:** Writing – review & editing, Visualization, Validation, Supervision.

## Declaration of competing interest

The authors declare that they have no known competing financial interests or personal relationships that could have appeared to influence the work reported in this paper.

## Figures and Tables

**Table:** 

**Hazard group**	**Reported climate hazards**
Biohazard	Vector borne disease
	Water borne disease
	Air borne disease
	Insect infestation
Chemical change	CO2 concentration
	Saltwater intrusion
Extreme temperature	Cold days
	Cold wave
	Winter conditions
	Heat wave
	Hot days
Flood	River flood
	Surface flood
	Coastal flood
	Permanent inundation
	Groundwater flood
Landslide	Landslide
	Rock fall
Storm	Rainstorm
	Severe wind
	Lightning thunder
	Tropical storm
	Fog
	Avalanche
	Cyclone
	Storm surge
	Heavy snow
	Monsoon
	Extra trop storm
Water scarcity	Drought
Wildfire	Forest fire
	Land fire

**Table:** 

**Action group**	**Adaptation actions**
Climate risk planning and management	Crisis management, including warning and evacuation systems
	Incorporating climate change into long-term planning documents
	Projects and policies targeted at those most vulnerable
Education, awareness, and engagement	Awareness campaign/education to reduce water use
	Public preparedness (including practice exercises/drills)
	Community engagement/education
	Diversifying power/energy supply
Flood management	Flood defenses − development and operation, storage
Hazard mapping and modelling	Flood mapping
	Heat mapping and thermal imaging
	Landslide risk mapping
	Sea level rise modelling
Health-related actions	Disease prevention measures
	Testing/vaccination programs for vector-borne disease
Increasing vegetation/greenspace coverage	Soil retention strategies
	Green roofs/walls
	Tree planting and/or creation of greenspace
Infrastructure improvement	Hazard resistant infrastructure design and construction
	Resilience and resistance measures for buildings
	Retrofit of existing buildings
	Maintenance/repair – leaking infrastructure
Initiatives to improve air quality	Air quality initiatives
Monitoring	Real time risk monitoring
	Biodiversity monitoring
Nature-based solutions	Nature based solutions
Temperature management	Shading in public spaces, markets
	White roofs
	Cooling systems for critical infrastructure
Urban planning	Restrict development in at risk areas
Water management	Storm water capture systems
	Promoting and incentivizing water efficiency
	Improve water supply distribution method
	Water butts/rainwater capture
	Additional reservoirs and wells for water storage
	Water efficient equipment and appliances
	Water use restrictions and standards
	Water use audits
	Xeriscapes – low water landscaping design
	Optimizing delivery fuel mix of water supply
	Water smart metering
	Diversification of water supply
**No action**	**No action currently taken**

**Table:** 

**Challenge groups**	**Challenges to adaptation action**
Access to quality/relevant data	Access to quality/relevant data
Community engagement	Community engagement
Financial/resource availability	Budgetary capacity
	Resource availability
	Economic diversity
	Economic health
Migration	Migration
Public health	Public health
Safety and security	Safety and security
Economic	UnemploymentInequality
Governance	Political engagement/transparency
	Government capacity
	Political stability
Living conditions	Housing
	Cost of living
	Poverty
Education and services	Access to education
	Access to basic services
	Access to healthcare
Urban environment and development	Rapid urbanization
	Environmental conditions
	Land use planning
	Infrastructure conditions/maintenance
	Infrastructure capacity

**Table:** 

			**Number of cities reporting hazard, by country**							
**Hazard**	**Total reports (# cities)**	**% of cities reporting hazard**	**Argentina**	**Brazil**	**Chile**	**Colombia**	**Costa Rica**	**Guatemala**	**Mexico**	**Peru**
Water scarcity − Drought	55	44	4	21	5	7	−	1	9	8
Ext. prec − Rainstorm	50	40	5	21	2	5	2	3	9	3
Ext. temp − Heat wave	44	35	5	13	3	8	−	1	7	7
Mass movement − Landslide	37	30	−	17	2	7	1	2	6	2
Flood and sea level rise − Surface flood	35	28	2	14	3	5	1	2	6	2
BioHaz − Vector borne disease	35	28	2	17	2	2	1	2	7	2
Ext. Temp − Hot days	34	27	2	16	3	2	−	1	6	4
Storm and wind − Severe wind	30	24	2	17	2	3	−	2	3	1
Flood and sea level rise − River flood	29	23	1	13	1	5	−	3	5	1
Wild fire − Forest fire	25	20	1	9	2	6	−	−	6	1
Chem. Change − CO2 concentration	17	14	−	9	1	3	−	1	1	2
Flood and sea level rise − Coastal flood	15	12	−	8	1	1	−	1	3	1
BioHaz − Water borne disease	13	10	−	7	−	2	−	1	3	−
Wild fire − Land fire	12	10	−	4	4	2	1	1	−	−
Storm and wind − Lightning thunder	8	6	1	2	1	−	−	1	2	1
Storm and wind − Tropical storm	8	6	1	4	−	1	−	−	2	−
BioHaz − Air borne disease	7	6	−	3	−	2	−	−	2	−
BioHaz − Insect infestation	7	6	−	5	−	−	−	1	1	−
Mass movement − Rock fall	6	5	−	2	−	−	−	1	3	−
Ext. temp − Cold days	6	5	1	−	2	−	−	1	2	−
Ext. prec − Fog	6	5	1	4	−	−	−	−	−	1
Ext. temp − Cold wave	5	4	−	−	1	1	−	−	1	2
Storm and wind − Avalanche	4	3	−	1	−	−	−	1	1	1
Storm and wind − Cyclone	3	2	1	1	−	−	−	−	1	−
Storm and wind − Storm surge	3	2	−	2	−	−	−	−	1	−
Ext. temp − Winter conditions	3	2	−	1	−	1	−	1	−	−
Ext. prec − Heavy snow	3	2	−	−	−	1	−	1	1	−
Ext. prec − Monsoon	3	2	1	−	−	−	−	1	−	1
Flood and sea level rise − Permanent inundation	2	2	−	2	−	−	−	−	−	−
Flood and sea level rise − Groundwater flood	2	2	−	1	−	−	−	−	−	1
Mass movement − Subsidence	2	2	−	−	−	−	−	−	2	−
Chem. change − Saltwater intrusion	2	2	−	−	1	1	−	−	−	−
Storm and wind − Extra trop storm	1	1	−	1	−	−	−	−	−	.

## Data Availability

Data will be made available on request.

## Appendix A . Response grouping of climate hazards, adaptation actions, and challenges to adaptation

**Climate hazards reported by Latin American cities**Note: Reported climate hazards correspond to response options within CDP Cities 2019 Questionnaire. Hazard groupings were informed by literature review and consultation with CDP representatives.

**Adaptation actions reported by Latin American cities**Note: Actions correspond to response options within CDP Cities 2019 Questionnaire. Groupings were informed by literature review and consultation with CDP representatives. Some actions may apply to multiple action groups; we assigned each action to the most directly relevant category.

**Challenges to adaptation action reported by Latin American cities**Note: Challenges to adaptation correspond to response options within CDP Cities 2019 Questionnaire. Groupings were informed by literature review and consultation with CDP representatives.

## Appendix B

Climate hazards reported by CDP cities in SALURBAL countries and percentage of cities reporting climate hazards by country in 2019
